# Clustering by Plasma Lipoprotein Profile Reveals Two Distinct Subgroups with Positive Lipid Response to Fenofibrate Therapy

**DOI:** 10.1371/journal.pone.0038072

**Published:** 2012-06-12

**Authors:** Kees van Bochove, Daniël B. van Schalkwijk, Laurence D. Parnell, Chao-Qiang Lai, José M. Ordovás, Albert A. de Graaf, Ben van Ommen, Donna K. Arnett

**Affiliations:** 1 Department of Microbiology and Systems Biology, TNO, Zeist and Leiden, The Netherlands; 2 Analytical Sciences Division, The Leiden Amsterdam Centre for Drug Research, Leiden, The Netherlands; 3 The Netherlands Bioinformatics Centre (NBIC), Nijmegen, The Netherlands; 4 The Nutrition and Genomics Laboratory, JM-USDA Human Nutrition Research Center on Aging at Tufts University, Boston, Massachusetts, United States of America; 5 Department of Epidemiology, University of Alabama at Birmingham, Birmingham, Alabama, United States of America; Governmental Technical Research Centre of Finland, Finland

## Abstract

Fibrates lower triglycerides and raise HDL cholesterol in dyslipidemic patients, but show heterogeneous treatment response. We used k-means clustering to identify three representative NMR lipoprotein profiles for 775 subjects from the GOLDN population, and study the response to fenofibrate in corresponding subgroups. The subjects in each subgroup showed differences in conventional lipid characteristics and in presence/absence of cardiovascular risk factors at baseline; there were subgroups with a low, medium and high degree of dyslipidemia. Modeling analysis suggests that the difference between the subgroups with low and medium dyslipidemia is influenced mainly by hepatic uptake dysfunction, while the difference between subgroups with medium and high dyslipidemia is influenced mainly by extrahepatic lipolysis disfunction. The medium and high dyslipidemia subgroups showed a positive, yet distinct lipid response to fenofibrate treatment. When comparing our subgroups to known subgrouping methods, we identified an additional 33% of the population with favorable lipid response to fenofibrate compared to a standard baseline triglyceride cutoff method. Compared to a standard HDL cholesterol cutoff method, the addition was 18%. In conclusion, by using constructing subgroups based on representative lipoprotein profiles, we have identified two subgroups of subjects with positive lipid response to fenofibrate therapy and with different underlying disturbances in lipoprotein metabolism. The total subgroup with positive lipid response to fenofibrate is larger than subgroups identified with baseline triglyceride and HDL cholesterol cutoffs.

## Introduction

Fibrates are prescribed to lower plasma triglycerides and raise HDL cholesterol (HDLc) in dyslipidemic patients. These drugs are not universal in efficacy; patients respond heterogeneously, and the most recent data show that fibrates only reduce cardiovascular events in specific subgroups [1]–[3]. It is therefore important to find improved methods for identifying those patients that will respond positively to fibrate treatment.

The fact that patients respond heterogeneously to fibrates has been documented in most of the large clinical trials conducted with fibrates [4]–[11]. Subgroups with high triglycerides [8], [12], high HDLc [12], a combination of high triglycerides with low HDLc or a combination of high triglycerides with a high LDLc/HDLc ratio [12], [13] and diabetic or insulin resistant subjects [14] all proved to have increased benefit from fibrates in specific studies, when compared to the general population. Because different studies use different methods for defining subgroups, apparently it is a challenge to optimally define the patient subgroup which stands to see the greatest benefit.

Methods to effectively define this high-benefit subgroup would be useful for analyzing finished trials and possibly for designing future clinical trials [15]. In earlier fibrate trials [4]–[11], owing to the heterogeneous response to therapy, the benefit of fibrates could not be shown over the whole population. A method to effectively identify the responding subgroup would both ensure that the right patients are treated with fibrates, and that clinical trials have the largest possible power to detect treatment benefit by appropriate selection of study participants. In trials that have already been conducted, correctly identifying the response subgroup would increase the power of subgroup analysis. For patients, it would mean a better opportunity to receive efficacious medication. Effective subgroup identification therefore would help patients, the pharmaceutical industry and healthcare economics.

Detailed measurements of the plasma lipoprotein profile [16], [17] should provide the basis for improving the patient-subgroup definition for treatment response to fibrates. Good response to fibrates has often been associated with an ‘atherogenic lipoprotein phenotype’ or ‘lipid triad’, which consists of raised triglycerides, low HDLc and small-sized LDL particles [18]. Fibrates activate transcription factors called peroxisome proliferator-activated receptors (PPARs) [19], [20]. This results in increased lipolysis of VLDL particles, increased removal of LDL particles and increased HDL production [21]. Fibrates, therefore, contribute substantially to the improvement of the atherogenic lipoprotein phenotype [22]. A detailed plasma lipoprotein profile contains information about the number of particles in subclasses of HDL, LDL and VLDL, and thus reflects more aspects of this phenotype than any routine clinical chemistry measurement. In addition, we have developed the Particle Profiler model [23], [24] to analyze lipoprotein profiles and detect metabolic disturbances, especially in the VLDL region through the VLDL performance parameter [25]. Therefore, lipoprotein profiles contain much information and hold great potential for improving the definition of fibrate response subgroups.

**Figure 1:**
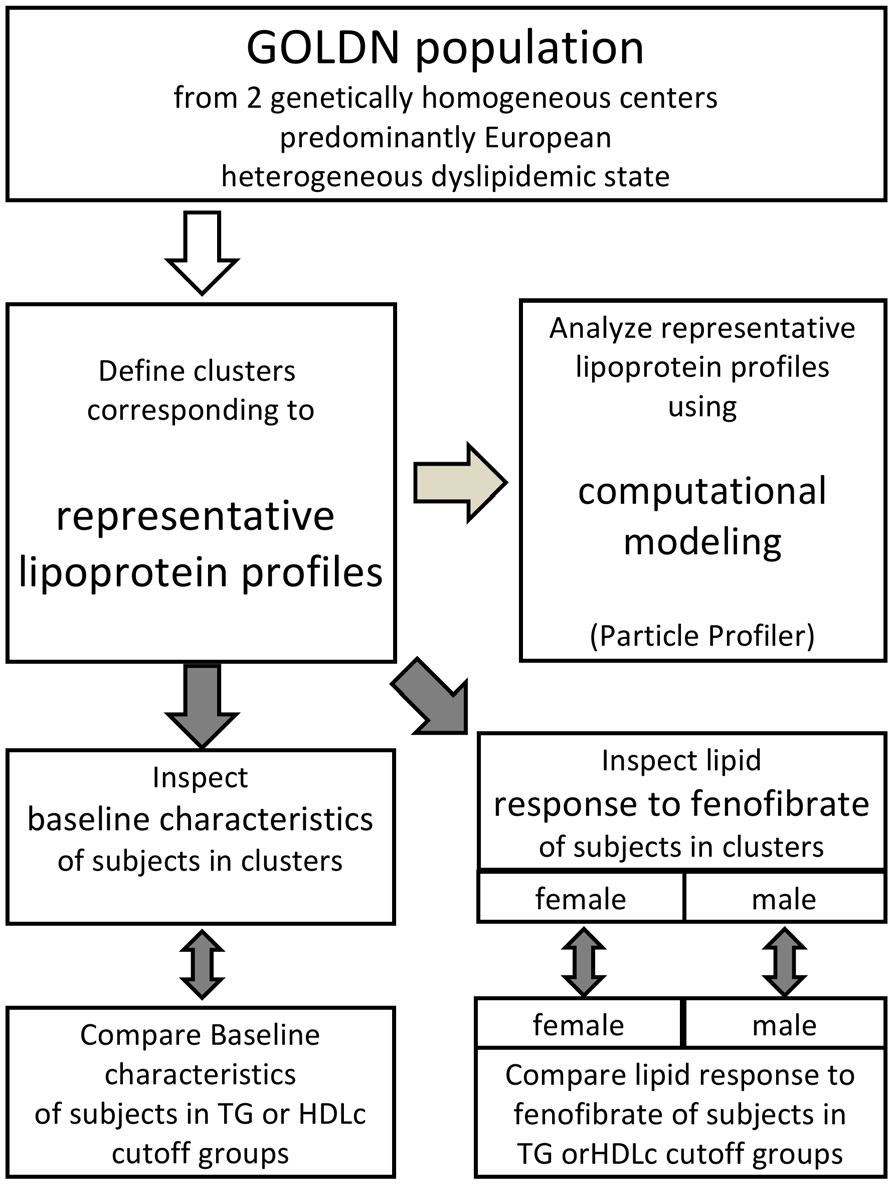
Overview of the data analysis approach presented in this paper. Clustering was carried out to identify representative lipoprotein profiles. The computational model analyzed those representative lipoprotein profiles. In the corresponding subgroups baseline characteristics and the lipid response to fenofibrate intervention was studied. The results of the subgroup studies were compared to the baseline characteristics and lipid response to fenofibrate in subgroups identified using triglyceride or HDL cholesterol cut-off methods.

From the above we can conclude that it is useful to define the relationship between baseline lipoprotein profiles and fibrate treatment response. Therefore, in this study (*see* Figure 1) we have used a clustering methodology to delineate subgroups of subjects with baseline lipoprotein profiles that are representative for the variation in the population at baseline. We then applied Particle Profiler to the representative ‘centroid’ lipoprotein profiles to investigate the metabolic disturbance associated with these representative profiles. Finally, we investigated whether subgroups based on the baseline lipoprotein profile clustering segregate the lipid response to fenofibrate treatment differently than subgroups based on triglyceride or HDLc cutoffs, as used for previous subgroup analyses [8], [12]. For this purpose, we have used data from the Genetics of Lipid Lowering Drugs and Diet Network (GOLDN) study [26], in which fenofibrate was administered to assess the variable response of triglyceride lowering in a white population recruited at two genetically homogenous centers with a very heterogeneous degree of dyslipidemia.

**Table 1:** Baseline characteristics of subjects in clustering on lipoprotein profile.

	cluster 1 (n = 350)	cluster 2 (n = 297)	cluster 3 (n = 128)
Age	46±17	50±15†	55±14† ‡
BMI	26.8±5.8	29.9±5.2†	30.0±3.8†
Female	67%	39%	34%
Total TG (mg/dL)	90±43	134±55†	290±129† ‡
Postprandial TG (t = 3 hours, mg/dL)	172±82	260±105†	477±178† ‡
Postprandial TG (t = 6 hours, mg/dL)	146±89	234±120†	513±239† ‡
Total cholesterol (mg/dL)	180±38	189±32†	223±40† ‡
LDLc (mg/dL)	114±31	129±27†	137±34† ‡
HDLc (mg/dL)	54±13	42±9†	36±7† ‡
LDL size (nm)	21.5±0.5	20.3±0.5†	19.7±0.4† ‡
LDLp (nmol/L)*	1106±301	1551±346†	1936±486† ‡
HDLp (nmol/L)*	31±5	30±6†	28±6† ‡
HOMA	2.9±2.1	4.1±2.8†	4.9±3.1†
CRP	0.2±0.4	0.3±0.3	0.2±0.2
Glucose (mg/dL)	97±16	104±16†	110±26† ‡
Drinkers	49%	42%	52%
Smokers	29%	30%	38%
Prior use of lipid-lowering agents	13%	19%	44%
Metabolic syndrome (ATP III)	19%	47%	88%
Diabetes	5%	9%	13%
Hypertension	22%	27%	36%

† indicates significantly different with respect to cluster 1, p<0.01.

‡ indicates significantly different with respect to cluster 2, p<0.01.

* LDL/HDL particle number (measured by NMR).

## Methods

### Study Sample

In the GOLDN study, family members from three-generational pedigrees were re-recruited from two centers of the ongoing NHLBI Family Heart Study (Salt Lake City and Minneapolis). The population can be described as from two genetically homogeneous centers (predominantly white), and encompassing a wide distribution in terms of lipid phenotypes. The only lipid inclusion criterion used was fasting TG <1500 mg/dL. The population is highly interrelated; subjects in the present sample represent 165 different families. The dyslipidemic state of the subjects is highly heterogeneous.

The subjects underwent a 3-week treatment with a daily dose of 160 mg fenofibrate. Before and after the intervention, lipid phenotypes were measured using both biochemical measurements and NMR spectroscopy, after fasting and postprandially after a dietary fat challenge (as described in [26]). Glucose and insulin were also measured, and used to calculate the HOMA index for insulin sensitivity [27]. In the present study, only fasting data are used. Details regarding the study and protocols followed can be found in Lai *et al.* [26]. Written informed consent was obtained from each participant at his/her screening visit. The GOLDN protocol and data handling were approved by the Institutional Review Boards at the University of Minnesota, the University of Utah, University of Alabama at Birmingham and Tufts University. Adverse events were monitored, and all incidents were reviewed by the local Principal Investigator, with any serious adverse events reported to the Institutional Review Boards at the University of Minnesota or the University of Utah and the NHLBI. The sample for this study consists of all 775 participants in GOLDN who had a complete NMR lipoprotein profile recorded both at baseline and after fenofibrate intervention.

**Figure 2:**
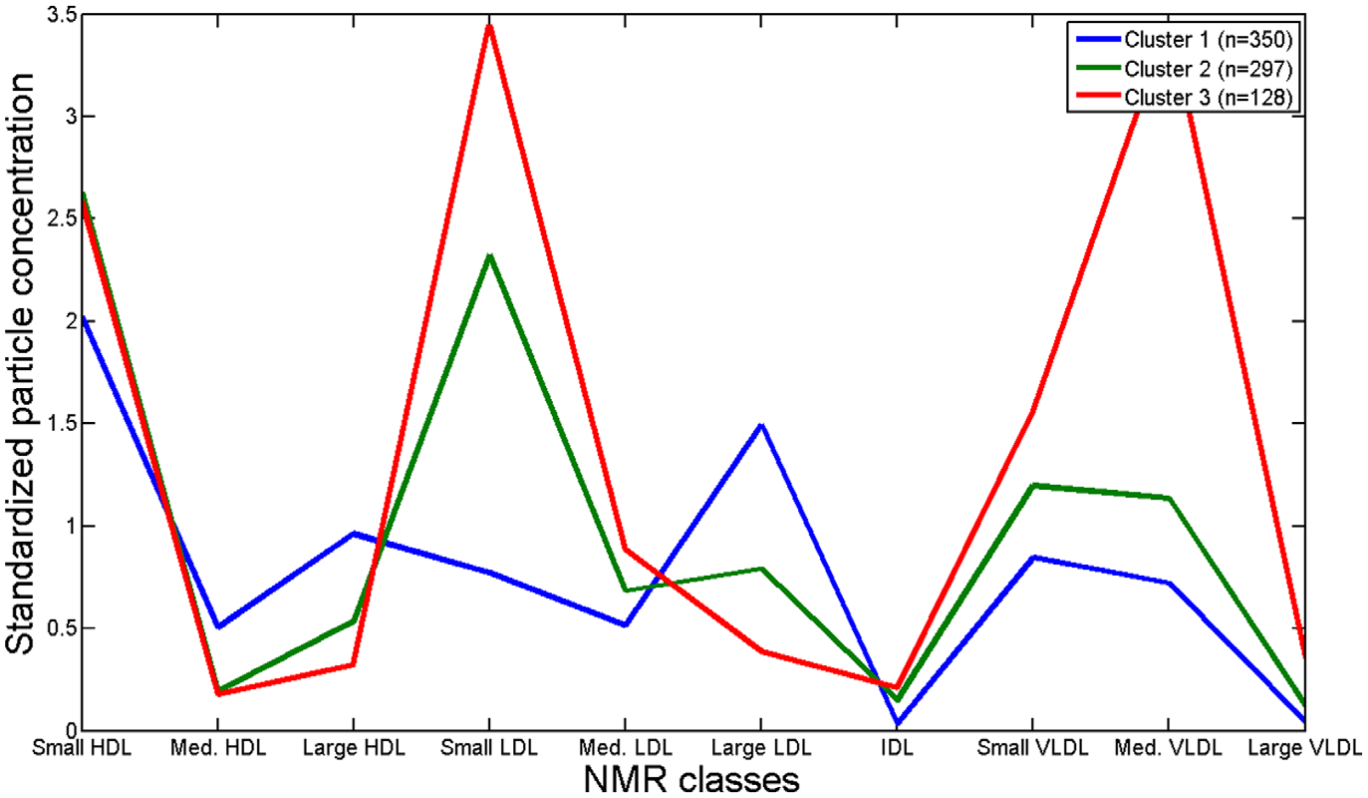
Mean standardized particle concentrations (unitless) of NMR lipoprotein subclasses in three subgroups based on K-means clustering. Particle sizes of the various subclasses were the same as described in Freedman, *et al.* [38].

**Figure 3:**
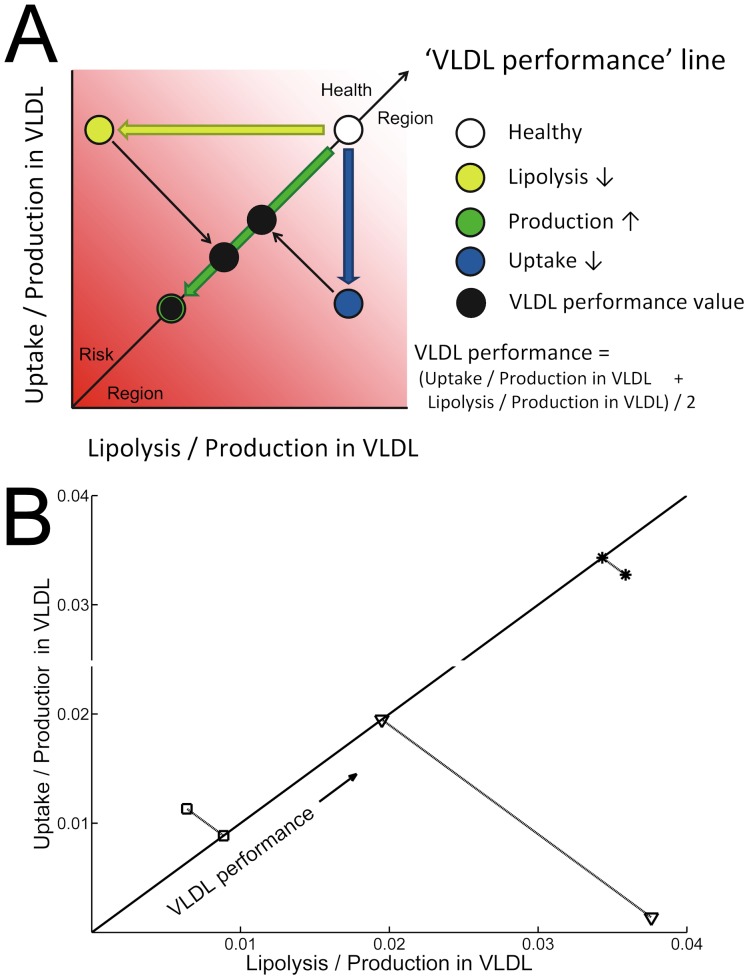
VLDL metabolism status as derived by Particle Profiler from the three representative ‘centroid’ lipoprotein profiles shown in Figure 2. A: Graphical representation of the VLDL performance diagnostic, from [25]. When applying the Particle Profiler model to a lipoprotein profile, the uptake/production and lipolysis/production ratios in VLDL can be quantified. The information from these ratios can be summarized in a single statistic taking the mean of these two ratios, which can be visualized as a projection on the identity line. We propose the name VLDL performance for this projection. It integrates information about production, lipolysis and uptake rates, but can be calculated without metabolic flux information, based on one detailed lipoprotein profile measured in one fasting blood sample. B: The analysis results. Cluster 1 (*) has a high VLDL performance, cluster 2 (▾) a lower VLDL performance, mainly due to hepatic uptake dysfunction, and cluster 3 (□) has an even lower VLDL performance, where the further lowering is especially due to impaired extrahepatic lipolysis.

### Subgroup Identification

We applied K-means clustering based on baseline lipoprotein profile data to identify representative lipoprotein profiles. We analyzed these lipoprotein profiles using computational modeling to give insight into representative dyslipidemic states. We also studied the baseline characteristics and response to fenofibrate of these subgroups (*see* Figure 1). By working with baseline lipoprotein profiles measured in fasting state, we ensure that the subgroups identified here can be used in clinical practise, if they result useful. An NMR measurement on a fasting blood sample is more feasible in clinical practise than on a standardized postprandial sample. So we use fasting NMR lipoprotein profiles as input to the K-means clustering routine to ensure that application of our results in the clinic is feasible.

K-means clustering is an unsupervised classification method which partitions a dataset into K non-overlapping clusters. First, K centroids were defined by randomly choosing K different data points, after which each data point was assigned to the nearest centroid. We used squared Euclidean distance as distance measure. Next, the cluster centroids were iteratively updated until the sum of the distances between the data points and the centroid in each cluster was minimal. This method was used on the baseline lipoprotein profile of all subjects to identify subgroups in the population. This profile was measured by NMR spectroscopy as described [16], [28], [29], and particle concentrations from three VLDL (large, medium and small), four LDL (IDL, large, medium small and very small) and three HDL (large, medium and small) subclasses were used, thus providing a 10-dimensional data space for clustering. To ensure that the classes were in the same range, the data were normalized: the values were divided by the total standard deviation in the VLDL, LDL and HDL classes respectively. To improve reproducibility, clustering was replicated 500 times, and the outcome with lowest total sum of distances was chosen. The resulting clusters were then sorted on size, such that the largest cluster was labeled cluster 1. For the used dataset, this procedure yields a reproducible final result.

We chose K = 3 because it led to distinct lipoprotein profiles that represent the variation in the population. At K = 3 both the LDL and VLDL range major variations in the concentration were captured, while the means and standard deviations of the clusters remained fairly separated (*see* Fig 1). The third added cluster is therefore an improvement over K = 2, since we can identify an additional representative lipoprotein profile. With K>3, the means and standard deviations of two of the cluster centroids became very similar, indicating no real difference in representative lipoprotein profiles. Moreover, at K = 3 baseline characteristics were clearly different between the three resulting groups. With K>3 some groups overlapped in important baseline characteristics such as LDL cholesterol concentration. When using the clustering approach, we do not expect the data to be perfectly separable into these three clusters. Indeed, other cut-off methods define a cut-off value that separates the population into two groups, but the separation is usually not naturally present in the data. In the same way, we do not expect a natural separation in the population based on the representative lipoprotein profiles. Still, the clustering is able to identify representative lipoprotein profiles and define ‘multivariate cut-off groups’ based on these profiles.

Clustering was performed on baseline NMR profiles of all 775 subjects, men and women together. In this way we were able to study sex differences to fenofibrate response in subgroups with the same representative lipoprotein profile. Furthermore, grouping on TG (low: TG <150, medium: 150≤ TG <200, and high: TG ≥200 mg/dL) and on HDLc (low: HDLc <40 mg/dL for males and HDLc <50 mg/dL for females) was implemented. Although total TG and total LDL and HDLc were measured by NMR as well, we used the biochemically measured values throughout this study to facilitate comparison with other studies. Biochemically and NMR-measured TG are highly correlated (R^2^ = 0.97); cholesterol measurements were less correlated (the lowest correlation being R^2^ = 0.80 for LDL cholesterol).

### Modeling Lipoprotein Profiles

We applied the previously developed Particle Profiler model [23] to the three representative ‘centroid’ NMR lipoprotein profiles. Particle Profiler was developed from the idea that although a single lipoprotein profile does not contain any lipoprotein metabolic flux information, it is still possible to derive ratios of metabolic flux parameters if the profile is carefully analyzed. The model can derive these ‘lipoprotein metabolic ratios’ by learning from earlier lipoprotein metabolic flux studies how the metabolic rate constants depend on the size of the lipoprotein particle [25]. Once the model has incorporated this knowledge, it can analyze lipoprotein profiles and determine the ‘lipoprotein metabolic ratios’ for each measured profile. In this study, we used the lipoprotein profiles to derive the ratio between uptake and production in VLDL, the ratio between lipolysis and production in VLDL, as well as the average of these last two ratios, which we have called ‘VLDL performance’ [25].

**Figure 4:**
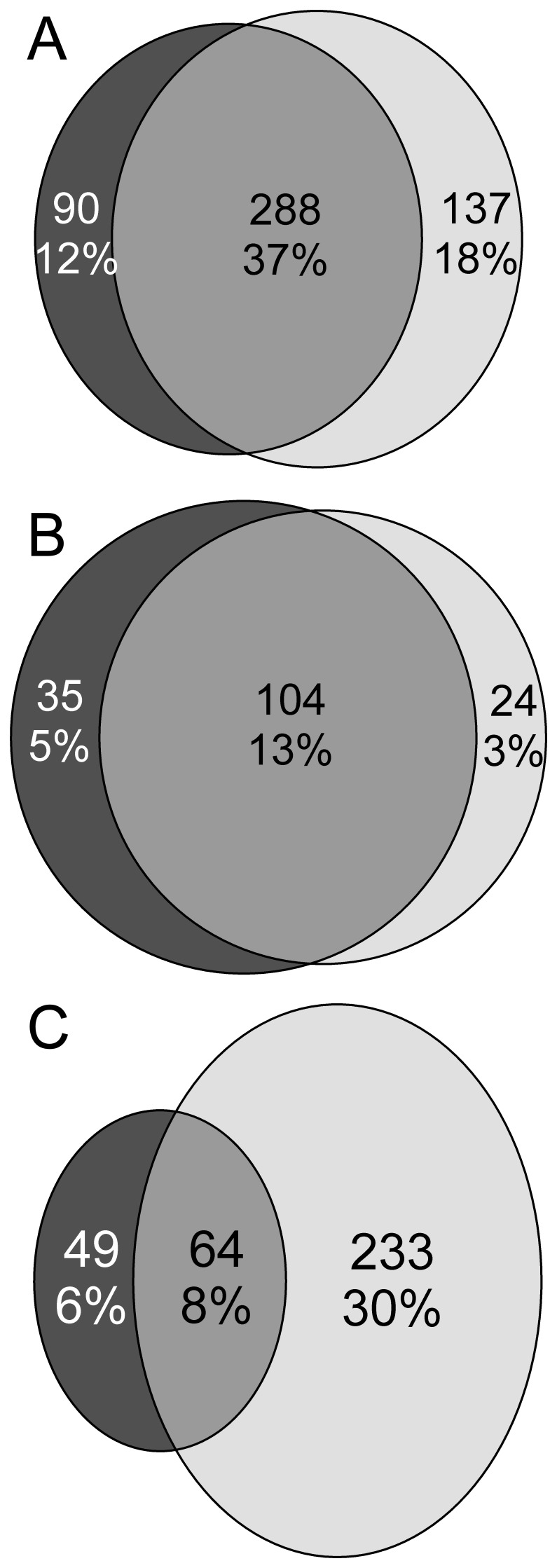
Subject overlaps between different subgroup identification methods. A: Subject overlap between the low HDLc subgroup (dark circle) and the sum of lipoprotein profile-based cluster 2 and 3 (light circle). Figures indicate the number of subjects in each group, in absolute numbers and as percentage of the total study population. **B:** Subject overlap between the high baseline-triglyceride subgroup (dark circle) and lipoprotein profile-based cluster 3 (light circle). Figures indicate the number of subjects in each group, in absolute numbers and as percentage of the total study population. **C:** Subject overlap between the medium baseline-triglyceride subgroup (dark circle) and lipoprotein profile-based cluster 2 (light circle). Figures indicate the number of subjects in each group, in absolute numbers and as percentage of the total study population. Lipoprotein cluster 2 clearly identifies a larger group of fibrate responders than the medium baseline-TG group.

Since this is the first study in which Particle Profiler was fitted to NMR lipoprotein profiles, the following calibration steps needed to be taken. First, each measurement method can measure particle size slightly differently, the model requires a correction of the NMR particle size measurement, to compensate for these differences. This size correction was derived by fitting it to the three lipoprotein profiles simultaneously, as described in the File S2, resulting in a size correction *d_shift_*  =  −4.01 nm.

Second, because production fluxes are not known when fitting an NMR lipoprotein profile, these fluxes were fitted sequentially. We initially assumed the average production into the VLDL1, VLDL2, IDL and LDL classes reported by Packard et al [30], and used those to fit the lipolysis and uptake processes. Then we fixed lipolysis and uptake and fitted the production fluxes. We repeated these two steps until we achieved a stable fit of the lipoprotein particle concentrations. This process cannot produce accurate values for the lipolysis, uptake and production processes per se, but can accurately estimate the ratios of these processes, and the ratios are the information we want to derive. Finally, the fitting routine uses weights that determine the importance of deviations in each measured fraction. The weights are given and explained in the File S2 (numerical values in Supporting Table 4).

### Statistical Analysis

After defining subgroups via lipoprotein profile clustering, triglyceride and HDLc methods, we calculated the baseline characteristics and the response to fenofibrate intervention in the subgroups. We compared all continuous variables across subgroups by standard Student’s T-tests and reported differences as significant if p<0.01.

The response is defined as the percent change in biochemically measured TG, HDLc and LDLc, as well as in NMR measured LDL size, LDL particle concentration (LDLp) and HDL particle concentration (HDLp). To detect gender-specific responses, these numbers were calculated for men and women separately. Because the percent changes are distributed normally as judged by visual inspection of the histograms, the differences in response between groups were also tested for significance by Student’s t-test with p<0.01.

To compare the outcomes of the different subgrouping methods, Venn diagrams were drawn to visualize the overlap between corresponding subgroups. The response in those resulting groups was calculated and also tested for significant differences by Student’s t-test with p<0.01.

**Table 2:** Percent changes after fenofibrate intervention, grouped by NMR clustering.

	cluster 1 (n = 350)		cluster 2 (n = 297)		cluster 3 (n = 128)	
Gender	*Male (n = 115)*	*Female (n = 235)*	*Male (n = 182)*	*Female (n = 115)*	*Male (n = 84)*	*Female (n = 44)*
TG	−20% ± 27%	−28% ± 26%	−26% ± 27%	−30% ± 23%	−38% ± 22%† ‡	−44% ± 18%† ‡
VLDLp	−27% ± 45%	−35% ± 45%	−34% ± 34%	−39% ± 38%	−40% ± 23%	−53% ± 20%†
VLDL-TG	−26% ± 43%	−35% ± 43%	−32% ± 41%	−41% ± 32%	−46% ± 26%† ‡	−54% ± 21%† ‡
HDLc	3% ± 10%	6% ± 12%	8% ± 11%†	7% ± 13%	10% ± 13%†	17% ± 12%† ‡
HDLp*	3% ± 11%	5% ± 13%	8% ± 12%†	6% ± 13%	11% ± 19%†	17% ± 21%† ‡
LDLc	−21% ± 12%	−23% ± 14%	−12% ± 14%†	−17% ± 16%†	8% ± 21%† ‡	−7% ± 20%† ‡
LDLp*	−9% ± 14%	−5% ± 18%	−16% ± 14%†	−19% ± 15%†	−8% ± 26%‡	−17% ± 23%†
LDL size	−1.1% ± 2.8%	−2.4% ± 3.0%	2.5% ± 2.5%†	2.4% ± 2.9%†	4.0% ± 3.3%† ‡	4.3% ± 3.2%† ‡

† indicates significantly different with respect to cluster 1, p<0.01.

‡ indicates significantly different with respect to cluster 2, p<0.01.

* LDL/HDL particle number (measured by NMR).

## Results

### Clustering Subgroups

The clustering carried out on lipoprotein particle concentrations as measured by NMR spectroscopy, identifies three distinct lipid-metabolic phenotypes in the population (*see* Table 1). The first and largest cluster (45% of subjects) is characterized by low TG and HOMA values, normal BMI, and is two-thirds female. It is also the subgroup with the largest proportion of young people (68% of subjects below the age of 30 are in cluster 1). The second and third clusters, in contrast, have an overrepresentation of obese, high-triglyceridemic, male and insulin-resistant subjects. The main difference between clusters 2 and 3 lies in the TG concentration, which is caused by a much higher concentration of VLDL particles in cluster 3 (Figure 2). All clusters are separated very clearly by LDL size, which should be expected as LDL size is a linear combination of the concentrations of the LDL subclasses on which the clustering was partially based. LDL particles in cluster 1 are mainly concentrated in the large LDL subclass (21.2–23 nm), while in clusters 2 and 3 they are mainly in the very small LDL subclass (18–19.8 nm) [31].

### Modeling Results

There was a difference in VLDL performance (*see* Figure 3a) between the three lipoprotein profile clusters. As Figure 3b shows, cluster 1 had a high VLDL performance, cluster 2 had a lower VLDL performance and cluster 3 had the lowest VLDL performance. The difference between cluster 1 and 2 in Figure 3b has a larger changed component along the y-axis, while the difference between cluster 2 and 3 is mostly due to changes along the x-axis. As explained by figure 3a, this indicates that the difference between cluster 1 and 2 is more influenced by liver uptake dysfunction, while the difference between cluster 2 and 3 is more influenced by extrahepatic lipolysis dysfunction. Therefore, the three subgroups show differences in underlying lipoprotein metabolism.

**Table 3:** Percent changes after fenofibrate intervention in low HDLc subgroup versus subjects in lipoprotein clusters 2 and 3.

	cluster 2/3, not low HDL (n = 137)		cluster 2/3, low HDL (n = 288)		not cluster 2/3, low HDL (n = 90)	
Gender	*Male (n = 95)*	*Female (n = 42)*	*Male (n = 171)*	*Female (n = 117)*	*Male (n = 18)*	*Female (n = 72)*
TG	−31% ± 25%	−36% ± 24%	−29% ± 27%	−33% ± 22%	−20% ± 32%	−28% ± 23%
HDLc	7% ± 12%	6% ± 15%	10% ± 11%	11% ± 12%	6% ± 11%	11% ± 13%
HDLp*	7% ± 13%	4% ± 14%	10% ± 16%	11% ± 16%	0% ± 13%	7% ± 12%
LDLc	−11% ± 15%	−20% ± 16%	−2% ± 20%†	−12% ± 18%†	−16% ± 13%‡	−20% ± 14%‡
LDLp*	−16% ± 15%	−19% ± 16%	−12% ± 21%	−19% ± 18%	−11% ± 15%	−10% ± 15%† ‡
LDL size	2.6% ± 2.9%	1.2% ± 2.7%	3.2% ± 2.9%	3.6% ± 3.0%†	0.7% ± 2.4%† ‡	−0.5% ± 2.6%† ‡

† indicates significantly different with respect to cluster 2/3 and not low HDL subgroup, p<0.01.

‡ indicates significantly different with respect to cluster 2/3 and low HDL subgroup, p<0.01.

* LDL/HDL particle number (measured by NMR).

### Baseline Comparison with TG and HDLc Subgroups

The baseline characteristics of the subjects in the subgroups defined by TG and HDLc cutoffs are given in File S2, Supporting Table 1. The most striking difference between the lipoprotein profile clustering and the subgrouping is the subgroups’ size, which is visualized for the HDL subgroups in Figure 4a, and for the TG subgroups in Figures 4b and 4c. Although for the TG subgroups many characteristics are roughly comparable (cluster 1 matching the low, cluster 2 matching the medium and cluster 3 matching the high TG cutoff subgroup), cluster 2 is about 2.5 times larger than the medium TG subgroup.

The subgrouping on HDLc leads to two groups that are essentially independent of sex and age, while the TG subgrouping and certainly the lipoprotein profile clustering are correlated with age and gender. TG and HDLc concentrations and LDL size all differ significantly between all subgroups in all three methods. LDLc concentration and also HDL particle number is significantly different between all subgroups in the lipoprotein profile clustering, but not in the TG subgrouping.

### Results of the Fenofibrate Intervention

The results of the fenofibrate intervention, expressed as percent changes in primary lipid metabolites as well as in some NMR-specific measures, are summarized in Table 2. The changes are reported for all three implemented subgrouping methods: clustering on the NMR measured lipoprotein subclass concentrations (Table 2), cutoff on total baseline TG concentration (File S2, Supporting Table 2a), and a sex-dependent cutoff on baseline HDLc concentration (File S2, Supporting Table 2b).

#### Clustering subgroups

The three lipoprotein profile-based clusters each showed a different response to fenofibrate intervention. Subjects in cluster 3 had the largest decrease in triglycerides (both measured biochemically and by NMR) and for females also the largest increases in HDLc and particle number. LDL size was decreased in cluster 1, but by contrast increased in clusters 2 and 3. This was accompanied by a trend towards less LDLc lowering in these successive clusters. The LDL particle number decreased most in cluster 2, although among females this decrease was not significantly larger than in cluster 3. Because fenofibrate is primarily given to lower triglycerides, increase LDL size, and increase HDL, we saw the smallest benefit from fenofibrate treatment on lipid parameters in individuals in cluster 1, while clusters 2 and 3 showed an increasingly favorable response. Cluster 2 had the peculiarity of the highest LDL particle response, significantly so for male subjects. Fenofibrate treatment did not affect postprandial response differently in different subgroups.

#### Comparison with HDLc subgroups

Lipoprotein-profile based clusters identify an additional response subgroup with respect to the HDLc-based subgroups [12]. The overlap between the low baseline HDLc subgroup and lipoprotein clusters 2 and 3 taken together comprises 37% of the total population (*see* Figure 4a). Also, 12% of the population is included in the low HDLc subgroup, but not in lipoprotein clusters 2 and 3, while 18% of the population is included in lipoprotein clusters 2 and 3, and not in the low HDLc subgroup. Table 3 shows that those subjects included only in lipoprotein clusters 2 and 3 have a significantly larger LDL particle size increase, and in females also a larger LDL particle number increase, as a result of fenofibrate treatment, compared to the subjects only included in the low HDLc subgroup. Therefore, lipoprotein profile-clusters 2 and 3 taken together show a large overlap with the low HDLc subgroup, but also identify an important new subgroup of subjects with positive lipid response to fenofibrate treatment.

#### Comparison with TG subgroups

The lipoprotein-profile based clusters also identified an additional response subgroup with respect to the baseline triglyceride-based subgroups. The lipoprotein profile cluster 3 had a large overlap with the high-triglyceride subgroup; with only 3% of the total population being unique to the lipoprotein profile cluster and 5% of the total population being unique to the high triglyceride subgroup (*see* Figure 4 for overlaps and Table 4 for response to intervention). The main difference was found in cluster 2, in which the lipoprotein profile clustering included 30% of the total population that was not included in the medium triglyceride subgroup. In these two subgroups the response to fenofibrate of HDL and LDL parameters was similar (*see* Table 5). The response of triglycerides was more pronounced in the triglyceride-based medium risk subgroup. Therefore, we see that lipoprotein cluster 2 identified a large additional response subgroup, with a response to fenofibrate that was less pronounced for triglycerides, but similar for HDL and LDL parameters.

**Table 4:** Percent changes after fenofibrate intervention in high TG subgroup versus lipoprotein cluster 3.

	cluster 3, high TG (n = 104)		cluster 3, not high TG (n = 24)		not cluster 3, high TG (n = 35)	
Gender	*Male (n = 67)*	*Female (n = 37)*	*Male (n = 17)*	*Female (n = 7)*	*Male (n = 14)*	*Female (n = 21)*
TG	−41% ± 20%	−45% ± 18%	−27% ± 26%	−41% ± 18%	−53% ± 18%‡	−44% ± 17%
HDLc	10% ± 13%	18% ± 13%	11% ± 13%	13% ± 10%	9% ± 15%	7% ± 12%†
HDLp*	10% ± 18%	19% ± 21%	14% ± 22%	7% ± 18%	6% ± 12%	6% ± 15%
LDLc	11% ± 21%	−6% ± 17%	−5% ± 17%†	−12% ± 30%	−1% ± 16%	−17% ± 12%
LDLp*	−5% ± 28%	−16% ± 21%	−21% ± 10%	−24% ± 33%	−15% ± 21%	−15% ± 16%
LDL size	3.9% ± 3.4%	4.0% ± 3.1%	4.7% ± 3.2%	5.9% ± 3.6%	3.8% ± 3.0%	1.4% ± 4.0%†

† indicates significantly different with respect to cluster 3 and high TG subgroup, p<0.01.

‡ indicates significantly different with respect to cluster 3 and not high TG subgroup, p<0.01.

* LDL/HDL particle number (measured by NMR).

**Table 5:** Percent changes after fenofibrate intervention in medium TG subgroup versus lipoprotein cluster 2.

	cluster 2, medium TG (n = 64)		cluster 2, not medium TG (n = 233)		not cluster 2, medium TG (n = 49)	
Gender	*Male (n = 35)*	*Female (n = 29)*	*Male (n = 147)*	*Female (n = 86)*	*Male (n = 21)*	*Female (n = 28)*
TG	−37% ± 22%	−40% ± 19%	−23% ± 27%†	−27% ± 24%†	−29% ± 25%	−46% ± 17%‡
HDLc	7% ± 9%	4% ± 10%	9% ± 11%	8% ± 13%	10% ± 12%	12% ± 13%†
HDLp*	11% ± 14%	5% ± 12%	8% ± 12%	6% ± 13%	11% ± 22%	3% ± 17%
LDLc	−8% ± 15%	−16% ± 17%	−13% ± 14%	−17% ± 16%	−6% ± 17%	−19% ± 18%
LDLp*	−11% ± 17%	−18% ± 17%	−17% ± 14%	−20% ± 14%	−16% ± 13%	−13% ± 24%
LDL size	2.9% ± 2.7%	3.0% ± 2.8%	2.4% ± 2.5%	2.3% ± 2.9%	3.8% ± 3.8%	1.0% ± 4.2%

† indicates significantly different with respect to cluster 2 and medium TG subgroup, p<0.01.

‡ indicates significantly different with respect to cluster 2 and not medium TG subgroup, p<0.01.

* LDL/HDL particle number (measured by NMR).

### Classification of New Samples

The described K-means classification method is applicable only to a large sample group because of its unsupervised nature. To allow classification of new samples into the subgroups we report, we provide the resulting cluster centroids and standard deviations in File S2, Supporting Tables 3a and 3b. Samples that were measured via the same protocol and laboratory can be classified by dividing the subclass particle concentrations (in nmol/L) by the corresponding standard deviations (given in File S2, Supporting Table 3b; where IDL is seen as a LDL class), and calculating the squared Euclidean distance to all 3 centroids. Each sample is assigned to the cluster which has the lowest resulting distance. File S1 consists of an exel sheet that performs this calculation. It has been shown that the laboratory method employed here has a high degree of repeatability [26]. With this excel sheet, our clustering can directly be applied to clinical data. Whether the clusters we identified are indeed of clinical use, remains to be demonstrated using studies in which hard endpoints are included.

## Discussion

Earlier studies identified subgroups with different lipid response to fenofibrate treatment, based on baseline triglycerides [8], [12], HDLc or a combination of triglycerides with HDLc or with the LDLc/HDLc ratio [12], [13]. Detailed measurements of the plasma lipoprotein profile [16], [17] are good candidates for improving the subgroup definition for treatment response to fibrates and thus the clinical treatment protocol, as they contain much information on the atherogenic lipoprotein phenotype [18]. Therefore, we compared the lipid response to fenofibrate in lipoprotein profile-based subgroups to the response in HDLc-based and triglyceride-based subgroups.

First, we observed that lipoprotein profile clustering yielded subgroups with different metabolic risk profiles. In the ‘high risk’ cluster 3, subjects had higher body mass index, fasting glucose, HOMA index, LDLc, LDL particles, triglycerides, and lower LDL size, HDLc, and HDLp. In cluster 3 there were also significantly more subjects with metabolic syndrome according to the Adult Treatment Panel 3 guidelines [32] (*see* Table 1). As the lipid parameters and the HOMA index indicate more severe metabolic syndrome in the higher risk subgroup, we conclude that the lipoprotein-based subgroups reflect differences in metabolic health. We also see that the higher risk subgroups were more likely to have been on lipid-lowering agents prior to the study, which is a further indicator of metabolic disease. The modeling results show decreasing ability to accommodate produced VLDL particles when comparing ‘healthy’ cluster 1 to ‘high risk’ cluster 3. In addition, the model analysis indicates that the difference between cluster 2 and 3 is more due to extrahepatic lipolysis dysfunction than to liver uptake dysfunction. The difference between cluster 1 and 2 is, on the contrary, more due to liver uptake dysfunction than to extrahepatic lipolysis dysfunction. When Kleeman *et al*. [33] studied high-fat-diet induced insulin resistance in a time-resolved and tissue specific manner in mice, they found that the dyslipidemia and resulting insulin resistance first affected the liver and subsequently the adipose tissue. Our findings indicate that dyslipidemic lipoprotein metabolism dysfunction also seems to involve two stadia. The first affects liver function and the second affects extrahepatic lipolysis (involving fat tissue). It is striking that we find these stadia using an unsupervised clustering method, indicating that metabolic variations we observe are among the key causes of the variance of lipoprotein profiles within dyslipidemic heterogeneity of the GOLDN population.

From the response to fenofibrate in the different baseline lipoprotein profile clusters, we deduce that there is less benefit from fenofibrate treatment on lipid parameters in the subgroup with least metabolic syndrome at baseline. The medium and high risk subgroups both responded positively to fenofibrate, but in different ways. The high risk subgroup experienced the largest triglyceride lowering, LDL size increase and HDL increase while the medium risk subgroup had the largest LDL particle benefit.

We compared the fenofibrate response in lipoprotein clusters to the response in subgroups based on baseline HDLc or baseline triglycerides. When comparing to the low baseline HDLc subgroup, we saw that lipoprotein profile-clusters 2 and 3 taken together showed a large overlap with the low HDLc subgroup, but also identified a substantial new subgroup of fenofibrate responders. We saw that the high-risk lipoprotein cluster was very similar to the high baseline-triglyceride subgroup, but the medium-risk lipoprotein cluster identified a much larger response subgroup than the medium-triglyceride subgroup. The lipoprotein profile-based clusters, therefore, identified a large subgroup with positive lipid response to fenofibrate treatment, additional to both previous methodologies.

The differences in baseline characteristics and fenofibrate response between lipoprotein clusters were biologically plausible. Clusters 1, 2 and 3 successively had more VLDL, more and smaller LDL and less and smaller HDL particles in their average lipoprotein profiles. These traits are characteristic of metabolic syndrome. It was, therefore, not surprising that we found a larger degree of metabolic syndrome in successive clusters. The differences between clusters also helped to explain differences in fenofibrate response. Fibrates are PPAR-agonists [19], [20] and act to increase lipolysis of VLDL particles, to reduce cholesterol ester and triglyceride exchange between VLDL and HDL, to increase the removal of LDL particles, to increase HDL production and to stimulate reverse cholesterol transport [21]. As a result of these processes, fibrates decrease VLDL particle number, decrease LDL particle number, increase LDL size, and increase HDL particle number. In cluster 3, subjects exhibited abnormal values for all these traits, and, therefore, fenofibrate intervention could correct them all. In cluster 2, subjects had abnormal LDL and HDL traits, but normal VLDL particle number. Fenofibrate, accordingly, especially corrected the LDL and HDL abnormalities in these subjects. In cluster 1, there was even the supposedly adverse affect of a decrease in LDL size, caused by an increase in the concentration of small LDL particles. The modeling analysis pinpointed possible causes of the mentioned disturbances as progressively more related to extrahepatic lipolysis dysfunction compared to liver uptake dysfunction. The lipid response to fenofibrate indicates that fenofibrate can play a role in correcting both these dysfunctions. Hence, the differences in response between clusters can be understood mechanistically.

The current clustering is based on subjects in the GOLDN study, a genetically homogeneous, white population. Whether the same clusters are valid for other populations remains to be seen, but this approach is widely applicable. The fact that clusters reflect known lipoprotein abnormalities and the metabolic syndrome is encouraging. It would be interesting to perform this type of clustering with data from other fenofibrate trials [11], [15].

The additional responder subgroup is mainly identified in lipoprotein cluster 2, which we expect to show clinical benefit. Lipoprotein cluster 2 had a lower triglyceride response to fenofibrate than the medium baseline triglyceride subgroup. This smaller triglyceride lowering may be an obstacle to clinical benefit, because an analysis of the Veterans Affairs HDL Intervention Trial showed an increase of coronary heart disease events per 50-mg/dl increase of triglycerides at baseline [14]. On the other hand, the response of LDL particles in lipoprotein cluster 2 is at least as good as in the medium baseline-triglyceride subgroup, which may well indicate decreased risk [34], and also the decrease in HDLc [35] and increase in LDL size [36] in this cluster do indicate beneficial effects from fenofibrate. Whether this is sufficient to translate into fewer cardiovascular events remains to be demonstrated. Trials such as FIELD [11] and ACCORD [15], [37] may provide the data to test the clinical benefit.

We compared our lipoprotein profile-based subgroups to baseline triglyceride-based and HDLc-based subgroups. Other reports also used a combination of the high triglyceride cutoff with a HDLc cutoff or a LDLc versus HDLc ratio cutoff [12], [13]. Such a combination of cutoffs would take into account more risk factors, similar to a complete lipoprotein-profile approach. Yet, we did not include the comparison with these subgroups because our high-risk subgroup corresponded very well to the high triglyceride subgroup. Adding additional cutoffs to this high risk subgroup would narrow the subgroup, and make it less comparable to our high-risk subgroup. However, using additional cutoffs could be useful for defining an even higher-risk subgroup than that based on lipoprotein clustering alone. The primary use of lipoprotein clustering is to identify two responder subgroups, containing a larger number of responder subjects than identified using other subgrouping methods.

Although the lipoprotein profile-based groups do show differences in HOMA levels, the lipoprotein profile itself does not include information on the possible diabetic or insulin resistant states of subjects. This information is relevant because subgroup analysis of the VA-HIT study has demonstrated that lowering triglycerides with the fibrate gemfibrozil more effectively reduces cardiovascular events in diabetic subjects than in non-diabetic subjects [14]. Therefore, any subgrouping for cardiovascular risk based on lipoprotein profiles should always be supplemented with diabetes or insulin resistance information. Recent trials have only used type II diabetic patients [11], [37], and so provide an excellent opportunity for lipoprotein profile-based analysis.

Using this new lipoprotein profile clustering methodology, we have identified two distinct subgroups of subjects with positive lipid response to fenofibrate therapy. Modeling analysis suggests that the difference between the subgroups with low and medium dyslipidemia is influenced mainly by hepatic uptake dysfunction, while the difference between subgroups with medium and high dyslipidemia is influenced mainly by extrahepatic lipolysis disfunction. This is a new insight into the metabolic disturbances that underlie the variation in lipoprotein metabolism at the population level. The total number of identified responder subjects is larger than those based on previously reported baseline HDLc and triglyceride cutoffs. Our findings are key to the post-hoc analysis of large clinical trials such as FIELD [11] and ACCORD [37] because a larger subgroup with positive response translates into greater statistical power to show treatment benefit in that subgroup. Also, by employing lipoprotein profile diagnostics, more patients can benefit from fenofibrate treatment.

## Supporting Information

File S1 **Excel sheet for classifying new NMR samples into the clustering subgroups described in this paper.**
(XLS)

File S2 **Contains additional tables and details about the model fitting methods.**
(DOC)
