# Supplementary material for: Clustering by Plasma Lipoprotein Profile Reveals Two Distinct Subgroups with Positive Lipid Response to Fenofibrate Therapy
Source: PLoS One. 2012 Jun 12;7(6):e38072. doi: 10.1371/journal.pone.0038072 (PMC3373573; doi:10.1371/journal.pone.0038072)
Supplement: File S2 — Contains additional tables and details about the model fitting methods. (DOC) [file pone.0038072.s002.doc]

# Clustering by plasma lipoprotein profile reveals two distinct fenofibrate responder subgroups

# Supporting Information File S2

Kees van Bochove,^1,*^ Daniël B. van Schalkwijk,^1,2,3,*^ Laurence D. Parnell,^4^ Chao-Qiang Lai,^4^ José M. Ordovás,^4^ Albert A. de Graaf,^1^ Ben van Ommen,^1^ Donna K. Arnett^5^

^1^TNO Quality of Life, Business Unit Biosciences, Zeist and Leiden, the Netherlands; ^2^The Leiden Amsterdam Centre for Drug Research, Analytical Sciences division, Leiden, the Netherlands;

^3^ The Netherlands Bioinformatics Centre (NBIC);

^4^ The Nutrition and Genomics Laboratory, JM-USDA Human Nutrition Research Center on Aging at Tufts University, Boston, MA USA; ^5^The department of Epidemiology, University of Alabama at Birmingham

^*^ Authors contributed equally

Correspondence to Daniël B. van Schalkwijk, TNO Quality of Life, Business Unit Biosciences, PO box 360, 3700 AJ Zeist, the Netherlands. Email: daan.vanschalkwijk@tno.nl

Telephone: +31306944553

Telefax: +31306944986

**Abbreviated title:** Two Distinct Fenofibrate Responder Subgroups

**Supporting** Table 1a: Baseline characteristics TG cutoff

| **TG subgroup** | Low (n=523) | | Med. (n=113) | | High (n=139) | |
| --- | --- | --- | --- | --- | --- | --- |
| Age | 47 ± 17 |  | 53 ± 15 | † | 53 ± 14 | † |
| BMI | 27.7 ± 5.6 |  | 30.4 ± 5.5 | † | 30.1 ± 4.3 | † |
| Female | 53% |  | 50% |  | 42% |  |
| Total TG (mg/dL) | 96 ± 31 |  | 181 ± 28 | † | 306 ± 117 | †‡ |
| Total cholesterol (mg/dL) | 175 ± 36 |  | 201 ± 31 | † | 215 ± 41 | †‡ |
| LDL cholesterol (mg/dL) | 117 ± 30 |  | 137 ± 25 | † | 134 ± 33 | † |
| HDL cholesterol (mg/dL) | 49 ± 13 |  | 43 ± 10 | † | 37 ± 10 | †‡ |
| LDL size (nm) | 21.1 ± 0.7 |  | 20.3 ± 0.7 | † | 19.9 ± 0.6 | †‡ |
| LDL particles (nmol/L) | 1243 ± 370 |  | 1680 ± 418 | † | 1840 ± 474 | †‡ |
| HDL particles (nmol/L) | 31 ± 5 |  | 31 ± 7 |  | 29 ± 6 | †‡ |
| HOMA | 3.2 ± 2.5 |  | 4.4 ± 2.5 | † | 5.0 ± 3.1 | † |
| CRP | 0.2 ± 0.4 |  | 0.3 ± 0.4 |  | 0.2 ± 0.3 |  |
| Drinkers | 47% |  | 45% |  | 47% |  |
| Smokers | 28% |  | 35% |  | 35% |  |
| Taking lipid-lowering agents | 15% |  | 30% |  | 35% |  |
| Metabolic syndrome (ATP III) | 23% |  | 75% |  | 83% |  |
| Diabetes | 5% |  | 13% |  | 16% |  |
| Hypertension | 23% |  | 33% |  | 33% |  |

**Supporting Table** 1b: Baseline characteristics HDL cutoff

| **HDLc subgroup** | High (n=397) | | Low (n=378) | |
| --- | --- | --- | --- | --- |
| Age | 49 ± 16 |  | 49 ± 16 | † |
| BMI | 27.4 ± 5.5 |  | 29.7 ± 5.3 | † |
| Female | 52% |  | 50% |  |
| Total TG (mg/dL) | 116 ± 77 |  | 178 ± 108 | † |
| Total cholesterol (mg/dL) | 189 ± 41 |  | 183 ± 38 |  |
| LDL cholesterol (mg/dL) | 122 ± 31 |  | 125 ± 31 | † |
| HDL cholesterol (mg/dL) | 55 ± 12 |  | 37 ± 6 | † |
| LDL size (nm) | 21.2 ± 0.8 |  | 20.3 ± 0.7 | † |
| LDL particles (nmol/L) | 1285 ± 430 |  | 1548 ± 472 | † |
| HDL particles (nmol/L) | 33 ± 5 |  | 27 ± 5 | † |
| HOMA | 3.1 ± 2.1 |  | 4.3 ± 3.1 | † |
| CRP | 0.2 ± 0.4 |  | 0.2 ± 0.3 |  |
| Drinkers | 53% |  | 40% |  |
| Smokers | 30% |  | 31% |  |
| Taking lipid-lowering agents | 18% |  | 23% |  |
| Metabolic syndrome (ATP III) | 22% |  | 61% |  |
| Diabetes | 5% |  | 12% |  |
| Hypertension | 24% |  | 29% |  |

^†^ significantly different from first group, ^‡^ significantly different from second group

**Supporting Table 2a:** Response to fenofibrate intervention, grouped by TG cutoff

|  | **Low (n=523)** | | | | | | | | **Medium (n=113)** | | | | | | | | | **High (n=139)** | | | | | | | | | |
| --- | --- | --- | --- | --- | --- | --- | --- | --- | --- | --- | --- | --- | --- | --- | --- | --- | --- | --- | --- | --- | --- | --- | --- | --- | --- | --- | --- |
| **Gender** | *Male*  *(n=244)* | | | | *Female*  *(n=279)* | | | | *Male*  *(n=56)* | | | | *Female*  *(n=57)* | | | | | *Male*  *(n=81)* | | | | | *Female*  *(n=58)* | | | | |
| TG | -20% | ± | 26% |  | -25% | ± | 25% |  | -34% | ± | 24% | † | | -43% | ± | 18% | † | | -43% | ± | 20% | † | | -44% | ± | 18% | † |
| HDLc | 6% | ± | 11% |  | 7% | ± | 12% |  | 8% | ± | 10% |  | | 8% | ± | 12% |  | | 10% | ± | 13% | † | | 14% | ± | 14% | † |
| HDLp | 6% | ± | 12% |  | 6% | ± | 13% |  | 11% | ± | 18% | † | | 4% | ± | 14% |  | | 10% | ± | 18% |  | | 14% | ± | 20% | †‡ |
| LDLc | -17% | ± | 13% |  | -21% | ± | 15% |  | -7% | ± | 16% | † | | -17% | ± | 18% |  | | 9% | ± | 21% | †‡ | | -10% | ± | 17% | † |
| LDLp | -14% | ± | 14% |  | -9% | ± | 18% |  | -13% | ± | 16% |  | | -16% | ± | 20% | † | | -7% | ± | 27% | † | | -16% | ± | 19% | † |
| LDL size | 0.7% | ± | 3.1% |  | -1.4% | ± | 3.6% |  | 3.2% | ± | 3.2% | † | | 2.0% | ± | 3.7% | † | | 3.8% | ± | 3.3% | † | | 3.0% | ± | 3.6% | † |

**Supporting Table 2b:** Response to fenofibrate intervention, grouped by HDLc cutoff

| **HDLc subgroup** | **Low HDL (n=378)** | | | | | | | | | **High HDL (n=397)** | | | | | | | | | |
| --- | --- | --- | --- | --- | --- | --- | --- | --- | --- | --- | --- | --- | --- | --- | --- | --- | --- | --- | --- |
| **Gender** | *Male (n=189)* | | | | *Female (n=189)* | | | | | *Male (n=192)* | | | | | *Female (n=205)* | | | | |
| TG | -28% | ± | 27% |  | | -31% | ± | 23% |  | | -25% | ± | 26% |  | | -29% | ± | 26% |  |
| HDLc | 10% | ± | 11% |  | | 11% | ± | 13% |  | | 4% | ± | 11% | † | | 5% | ± | 12% | † |
| HDLp | 9% | ± | 16% |  | | 9% | ± | 15% |  | | 5% | ± | 12% |  | | 4% | ± | 14% | † |
| LDLc | -4% | ± | 20% |  | | -15% | ± | 17% |  | | -17% | ± | 15% | † | | -23% | ± | 15% | † |
| LDLp | -12% | ± | 21% |  | | -15% | ± | 17% |  | | -12% | ± | 15% |  | | -6% | ± | 19% | † |
| LDL size | 2.9% | ± | 2.9% |  | | 2.0% | ± | 3.5% |  | | 0.6% | ± | 3.5% | † | | -2.3% | ± | 3.3% | † |

^†^ significantly different from first group, ^‡^ significantly different from second group

**Supporting Table 3a: clustering centroids used**

| **Centroids** | Cluster 1 | Cluster 2 | Cluster 3 |
| --- | --- | --- | --- |
| Small HDL | 2.02019 | 2.62994 | 2.58552 |
| Medium HDL | 0.50396 | 0.19290 | 0.17742 |
| Large HDL | 0.96186 | 0.53607 | 0.32086 |
| Small LDL | 0.77363 | 2.32456 | 3.44883 |
| Medium LDL | 0.51607 | 0.68405 | 0.88327 |
| Large LDL | 1.49320 | 0.78965 | 0.38724 |
| IDL | 0.03379 | 0.14969 | 0.20903 |
| Small VLDL | 0.84682 | 1.19773 | 1.56219 |
| Medium VLDL | 0.72134 | 1.13719 | 3.46088 |
| Large VLDL | 0.04682 | 0.12658 | 0.36028 |

**Supporting Table 3b: standard deviations used**

| **Standard deviations** | |
| --- | --- |
| HDL | 9.019487 |
| LDL | 392.7842 |
| VLDL | 29.94605 |

## Fitting the NMR size correction

In this study, we sought to fit Particle Profiler to data from NMR lipoprotein profile measurements. This introduces the problem that different measurement methods may estimate the size of particles slightly differently. Our particle size-composition sub-model was based on measurements using the small-angle X-ray scattering (SAXS) method [1]. This size measure possibly differs from NMR size measurements [2], and therefore a size correction may well be necessary. This size correction cannot be calculated directly, and therefore needs to be estimated based on data. A direct calculation would be possible if we would have average particle composition data at different measured sizes for both methods. By comparing the particle composition, we could then derive the size shift between the two methods. However, in the case of NMR measurements, both the particle number and particle composition are calculated based on the NMR signal and not measured directly [3]. In this calculation, assumptions need to be made that can result in a slight discrepancy between biochemically measured values and NMR-measured values. This situation makes the particle composition data unsuitable for estimating the size shift, and only leaves the option of fitting the size shift $d_{shift}$ as a 'model constant', which is the same for all patients.

In order to fit the model constants, a two-layered fitting routine was constructed. On the first layer, the algorithm searched for the optimal value for the model constant. The second layer of the routine fitted the parameters of the model with each selected constant value, to the data of all subjects selected for estimating that constant. Both layers used the Levenberg-Marquardt algorithm as implemented in MATLAB's nlinfit method of version 7.7.0 (R2008b) for fitting the constants and parameters respectively. In this way, the model parameters were estimated per individual, while the model constants were estimated using a group of patients.

## Weights for fitting model to data

The NMR data in the GOLDN study were reported in 7 pools, shown in the table below. For fitting we used these pools, plus sums of the LDL through IDL and the VLDL ranges. Including those ranges in the fit is important to prevent systematic lowering or rising of the total modeled particle count compared to the measured particle count. The table also shows the weights these pools have been given for data fitting. The weights were determined with an eye to the importance that each pool has for determining the underlying processes. Especially the small and medium VLDL pools should really be fitted exactly right, since they are essential for a right characterization of the VLDL metabolism ratios. The largest VLDL pool was given less importance, as the model was developed with datasets of lipoprotein kinetics for which only two VLDL pools are available. To be able to interpret this large VLDL pool correctly, more detailed kinetics datasets are necessary, which are not available at this time. The large LDL and IDL pools are difficult to distinguish experimentally, and therefore we opted to fit these jointly. The individual subclass weights were set to zero, but they are taken into account in the IDL and LDL sum parameter. The LDL weights are systematically lower than the VLDL weights, this is done to correct for the average concentrations of these particles. There are generally far more VLDL than LDL particles in human plasma.

**Supporting Table 4: weights used for fitting the Particle Profiler model to NMR data**

| Fraction name | Weight |
| --- | --- |
| very small LDL | 0.0050 |
| medium small LDL | 0.0050 |
| large LDL | 0 |
| IDL | 0 |
| small VLDL | 0.1258 |
| medium VLDL | 0.1298 |
| large VLDL | 0.0641 |
| Sum very small LDL 🡪 IDL | 0.0065 |
| Sum small VLDL 🡪 large VLDL | 0.1702 |

Reference List

1. Tuzikov FV, Tuzikova NA, Galimov RV, Panin LE, Nevinsky GA (2002) General model to describe the structure and dynamic balance between different human serum lipoproteins and its practical application. Med Sci Monitor 8: MT79-88.

2. Otvos JD, Jeyarajah EJ, Bennett DW, Krauss RM (1992) Development of a proton nuclear magnetic resonance spectroscopic method for determining plasma lipoprotein concentrations and subspecies distributions from a single, rapid measurement. Clin Chem 38: 1632-1638.

3. Ala-Korpela M, Soininen P, Savolainen MJ (2009) Letter by Ala-Korpela et al Regarding Article, "Lipoprotein Particle Profiles by Nuclear Magnetic Resonance Compared With Standard Lipids and Apolipoproteins in Predicting Incident Cardiovascular Disease in Women". Circulation 120: e149.
